# A Scoping Review of Minimal Important Change and Minimal Detectable Change of the Fugl-Meyer Assessment Lower Extremity Scale in Patients with Stroke

**DOI:** 10.1298/ptr.E10324

**Published:** 2025-06-04

**Authors:** Tetsuharu NAKAZONO, Satoru AMANO, Kazuya SAITA, Kayoko TAKAHASHI

**Affiliations:** 1 Department of Rehabilitation, Kitasato University Hospital, Japan; 2 Department of Rehabilitation, School of Allied Health Sciences, Kitasato University, Japan; 3 Department of Psychosocial Rehabilitation, Graduate School of Biomedical and Health Sciences, Hiroshima University, Japan

**Keywords:** Treatment outcome, Stroke rehabilitation, Paresis, Reproducibility, Psychometrics

## Abstract

Objectives: In stroke rehabilitation, the Fugl-Meyer Assessment Lower Extremity (FMA-LE) motor scale is widely used to assess consecutive recovery steps from motor paralysis and predict prognosis after stroke. However, there has been limited opportunity to refer to the useful yet few studies that evaluated the minimal important change (MIC) and the minimal detectable change (MDC) of the FMA-LE motor scale. The aims of this review were to: (1) identify studies that have estimated the MIC and MDC, (2) identify the calculated MIC and MDC values and disease phases in previous studies, and (3) assess the methodological quality of the MIC and MDC studies of the FMA-LE motor scale. Methods: A scoping review was undertaken following the Preferred Reporting Items for Systematic reviews and Meta-Analyses (PRISMA) recommendations. 3 databases (PubMed, CINAHL, and Web of Science) were used for the literature search. Reports of the MIC and MDC of the FMA-LE motor scale in patients with stroke were selected. Results: 6 studies were included after confirming their eligibility. In the acute phase, inter-rater MDC was 3.23 points. In the early subacute phase, intra-rater MDC was 1.24 points. In the chronic phase, intra-rater MDC was 3.80, 4.87, and 7.98 points, inter-rater MDC was 3.57 and 5.96 points, and MIC was 6.00 points. Conclusion: No late subacute threshold was identified for the MDC, and no acute or subacute threshold was identified for the MIC. Compared with the quality of the MIC study, there is room for improvement in the quality of the MDC studies with respect to study design.

## Introduction

The use of appropriate outcome measures to interpret the clinical meaning of observed changes is important. Therefore, it is necessary to confirm appropriate psychometric values not only for validity, reliability, and responsiveness, but also for interpretability of the observed results^1)^. Interpretability includes many concepts, among which the minimal clinically important difference (MCID) has been continually emphasized for the interpretation of clinical research results^2,3)^. Recently, results are interpreted not only based on statistical significance, but also on whether there are clinically meaningful changes or differences.

The MCID was first defined in 1989 by Jaeschke et al^4)^. It has been used as a criterion for assessing the potential differences between beneficial effects for patients and statistical significance^5)^. The MCID is a promising concept, but there is no absolute consensus on the definitions or estimation methods^6)^. Anchor-based and distribution-based estimation methods have been used previously^4,7,8)^. The anchor-based MCID has been referred to by various terms, such as minimal important change (MIC), minimal important difference, clinically important difference, or clinically significant change^9–12)^. The distribution-based MCID has been referred to by various terms such as, minimal detectable change (MDC), minimal detectable difference, standard error of measurement, limit of agreement, or smallest detectable change^13–15)^. Therefore, in this study, we define the anchor-based method MCID as MIC and the distribution-based method MCID as MDC.

There are differences in the methods used for estimating the MIC^4,7,8,16)^. The MIC is based on patient scores or patient-reported outcomes (PROs)^4,7)^. For example, the Global Rating of Change Scales is a very popular anchor for calculating the MIC^17–19)^. Therefore, the MIC is believed to provide the best estimation of an individual’s perspective. However, it should be noted that the MIC does not contain information on measurement errors. Therefore, some researchers believe that investigating and comparing the results of other calculation methods that include information about measurement errors or reproducibility of the results^19–21)^ is necessary. These methods are often called distribution-based methods. They assess the distribution of reproducibility of the results and the MDC in scores among patients^7,22)^. It should be noted, however, that the MDC is inconsistent with clinicians’ intended purpose when they investigate clinical importance^23)^. The MDC defines only a clinically relevant indicator of “real” change based on the measurement error information. Therefore, investigating both MIC and MDC values is recommended^22)^.

Functional assessment is important for the diagnosis and prognosis of patients with disabilities. In the field of stroke rehabilitation, the Fugl-Meyer Assessment Lower Extremity (FMA-LE) motor scale is widely used to assess the consecutive steps of recovery from motor hemiparesis^24,25)^ and predict walking ability after stroke.^26)^ The scale has shown appropriate validity,^15,27–30)^ reliability,^15,25,27–30)^ and responsiveness^18)^ for clinical use. As a result, the FMA-LE motor scale has been used as an outcome in many rigorous clinical trials for patients with stroke^31–37)^. Similarly, the FMA-upper extremity (FMA-UE) motor scale is widely used to evaluate motor recovery in the upper extremity after stroke^24,38)^. The FMA-UE is frequently used in clinical trials as an outcome measure for upper extremity function^39)^. The psychometric properties of the FMA-UE have been extensively evaluated for use in clinical trials^24,40–42)^. Additionally, a systematic review of the MDC and MIC in the subacute and chronic phases has been conducted for the FMA-UE^43)^. However, although FMA-LE is clinically important, fewer outcome measures have been reported in clinical trials compared with FMA-UE^39)^. Because factors such as muscle strength, coordination, and balance are interrelated in lower extremity function, alternative indicators such as walking speed and independent walking are used in addition to FMA-LE^44–46)^. Moreover, recent trends in stroke rehabilitation research highlight a strong focus on upper extremity function. A review of large-scale randomized controlled trials (RCTs) published since 2014 identified that 12 out of 15 trials primarily investigated upper extremity function^39)^. This indicates a stronger research focus on upper extremity recovery, which may partly explain the limited number of studies calculating MDC and MIC for FMA-LE in clinical trials. Therefore, the results of studies that used the FMA-LE motor scale did not have a good opportunity to refer to MIC studies compared with the results of studies that used the FMA-UE motor scale due to there being only a few reports of the MIC of the FMA-LE motor scale^18)^. In addition, MIC information can be used as a reference only when the target patients have similar characteristics to the investigated patients (e.g., the MIC of patients with chronic stroke cannot apply to patients with subacute stroke^47,48)^). Therefore, there has been no comprehensive review specifically addressing MDC and MIC for FMA-LE, highlighting a gap in the existing literature.

The aims of this review were to (1) identify studies that have estimated the MIC and MDC, (2) identify the calculated MIC and MDC values and disease phases in previous studies, and (3) assess the methodological qualities of the MIC and MDC studies of the FMA-LE motor scale.

## Methods

This review was registered with the University Hospital Medical Information Network (UMIN) Clinical Trials Registry (UMIN000048612) as a pre-initiation condition. This review followed the Preferred Reporting Items for Systematic reviews and Meta-Analyses extension for Scoping Reviews (PRISMA-ScR) recommendations,^49)^ including Supplementary Material 1.

### Eligibility criteria

The inclusion criteria were as follows: (1) investigated the MIC and MDC of the FMA-LE motor scale for patients with stroke; (2) had implementation and scoring of the FMA-LE assessment by medical staff (e.g., physical therapists or occupational therapists) experienced in stroke rehabilitation; and (3) only English peer-reviewed journal articles were included to ensure methodological rigor and reduce the risk of bias. The exclusion criteria were as follows: (1) no abstract; (2) languages other than English; (3) review articles; (4) inclusion of patients with any other intracranial disease such as traumatic brain injury or brain tumour; (5) an unclear distinction from the FMA-UE motor scale; and (6) conference abstracts, doctoral dissertations, master's theses, and government reports were excluded due to the potential for limited methodological transparency and peer review.

### Information sources and search strategy

The databases searched were PubMed, Cumulative Index to Nursing & Allied Health Literature (CINAHL), and Web of Science. Literature searches were conducted from 1975, the year FMA was developed and published by Fugl-Meyer, to January 18, 2024. The search strategy was customized for each database and included three key concepts: the FMA, MIC, and MDC. A concrete search strategy for the three databases (i.e., PubMed, CINAHL, Web of Science) is shown in Supplementary Material 2. Additional search was conducted by hand searching. Reference lists of MCID previous study were also identified and hand-searched. The first author conducted a search on the Physiotherapy Evidence Database (PEDro) using the keyword “Fugl-Meyer Assessment.”

### Selection of sources of evidence

The results extracted from each database were exported to EndNote 20 software (Clarivate, Philadelphia, PA, USA), and duplicates were removed. Two reviewers (TN and KS) applied inclusion and exclusion criteria to all titles and abstracts during the screening phase to determine which articles should proceed to the full-text review (eligibility assessment) phase. In the eligibility assessment, the two reviewers independently selected the targeted articles with the eligibility criteria by full-text review. If the two reviewers disagree during the due eligibility assessment (25%), a third senior researcher (SA) made the final decision after reviewing the full text and hearing each reviewer’s opinions.

### Data charting process

All data were extracted from the included articles by two authors (TN and SA). The following information was extracted from each article: first author, year of publication, first author’s country, patients’ age, sex, type of stroke, time after stroke onset, disease phase, sample size, the FMA-LE total score, reliability information (simultaneous data or not, type, and intraclass correlation coefficient values), the MIC estimation approach, distribution-based results (values of the standard error of measurement and the MDC), and anchor-based results (type of anchor and the calculated value). The disease phases were determined based on the mean time after stroke onset in the subjects of the accepted papers, and were classified as acute (≤7 days from stroke onset), early subacute (≤7 days to 3 months from stroke onset), late subacute (≤3–6 months from stroke onset), or chronic (>6 months from stroke onset)^50)^.

### Risk of bias (quality) evaluation

The risk of bias and quality evaluations of the MIC and MDC information were performed using the COnsensus-based Standards for the selection of health Measurement INstruments (COSMIN) Risk of Bias checklist by two reviewers (TN and SA). In the current study, only three COSMIN checklists of reliability (9 items), measurement error (8 items), and responsiveness criterion approach (3 items) were used to evaluate the quality of the MIC and MDC studies. Each item was rated as “very good,” “adequate,” “doubtful,” “inadequate,” or “not applicable (NA).” Additionally, the risk of bias ratings was consistent between the 2 reviewers, indicating a high level of agreement in the evaluation process.

## Results

### Study selection

**Fig. 1.**
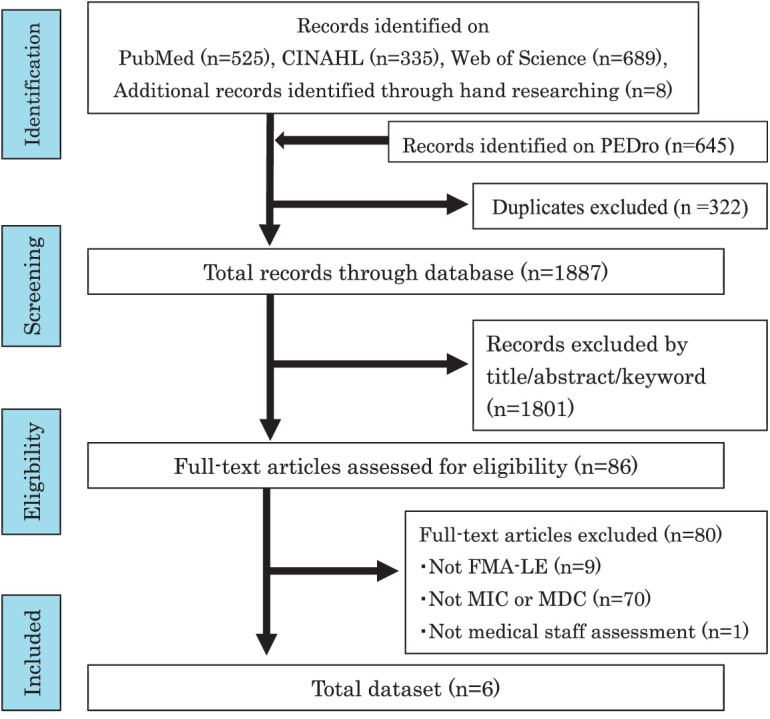
Flow chart of the literature search process. CINAHL, Cumulative Index to Nursing & Allied Health Literature; PEDro, Physiotherapy Evidence Database; FMA-LE, Fugl-Meyer assessment lower extremity; MIC, minimal important change; MDC, minimal detectable change.

A systematic search of PubMed (n = 525 articles), CINAHL (n = 335), and Web of Science (n = 689), PEDro (n = 645), Hand researched (n = 8) yielded a total of 2202 articles. After removing duplicates, 1887 articles were reviewed. Of these, 1801 publications were excluded because their titles and abstracts did not match the criteria. After that, 86 articles were reviewed for full-text papers. A total of 6 studies^15,18,27–30)^ were included in this systematic review. The reasons for exclusion were not investigating the FMA-LE (9 studies), not investigating the MIC and MDC (70 studies), and not performing the FMA-LE assessment by medical staff experienced in stroke rehabilitation (1 study)^51)^. In the research article excluded by the characteristics of implementers, the caregivers of the patients with stroke performed the FMA-LE under the remote instructions of the physical therapists, and the scoring system was changed; therefore, it is not included in the current study. Figure 1 outlines the process of study selection. The information datasets, which included all of the extracted data from each article, are summarized in Supplementary Material 3. Information needed for the data was obtained without contacting the authors.

### MIC information

The MIC values were presented by estimation method and disease phase. Regarding the MIC, a value of 6.00^18)^ was calculated in patients with chronic stroke using PROs (global rating of patient-perceived changes; GRPPC).

### MDC information

The MDC values were summarized based on the reliability study procedure (simultaneous or non-simultaneous data) and reliability type (intra-rater or inter-rater reliability) in Table 1. A simultaneous inter-rater MDC was reported from patients with chronic stroke. 1 study reported simultaneous inter-rater MDCs as 5.96^27)^ in the chronic stroke phase. 4 studies reported non-simultaneous intra-rater MDCs as 1.24^15)^ in the early-subacute stroke phase and 3.80,^28)^ 4.87,^30)^ and 7.98^27)^ in the chronic stroke phase. 2 studies reported non-simultaneous inter-rater MDCs as 3.23^15)^ in the acute stroke phase and 3.57^29)^ in the chronic stroke phase.

**Table 1.** Distribution-based methods estimation: minimal detectable change

Disease phase	MDC			
	Simultaneous		Non-simultaneous	
	Intra-rater	Inter-rater	Intra-rater	Inter-rater
Acute				3.23^15)^
Early subacute			1.24^15)^	
Late subacute				
Chronic		5.96^27)^	4.87^30)^	3.57^29)^
			7.98^27)^	3.80^28)^

Acute, ≤7 days from stroke onset; early subacute, ≤7 days-3 months from stroke onset; late subacute, ≤3–6 months from stroke onset; chronic, >6 months from stroke onset. Cells left blank indicate that MDC values were not reported or calculated for the corresponding condition. MDC, minimal detectable change.

### Assessment of the methodological quality of included studies.

To assess the quality of the 4 studies that used the MDC (n = 5^15,27–30)^), the COSMIN checklists of reliability and measurement error were used. Only one study^27)^ received a “very good” or “adequate” rating for more than half of the checklist items (when NA was excluded) for both checklists of reliability and measurement error. The details of the ratings for each item in the MDC studies are presented in Table 2 (reliability checklist) and Table 3 (measurement error checklist). To assess the quality of the study that used the MIC (n = 1^18)^), a COSMIN checklist of responsiveness-criterion approach was used. All checklist items were rated as “very good” or “adequate.” The details of the ratings for each item in the MIC study are presented in Table 4.

**Table 2.** Results of bias risk assessment of studies on reliability using COSMIN Risk of Bias

Study	Reliability type	Simultaneous	(1)	(2)	(3)	(4)	(5)	(6)	(7)	(8)	(9)
Beckerman et al.^30)^	Intra-rater	No	++	++	?	?	?	?	++	NA	NA
Hiengkaew V et al.^29)^	Inter-rater	No	?	?	?	?	?	?	++	NA	NA
Hsueh I et al.^28)^	Intra-rater	No	?	?	?	?	?	?	++	NA	NA
Kim H et al.^27)^	Intra-rater	No	?	++	?	++	++	?	++	NA	NA
Kim H et al.^27)^	Inter-rater	Yes	NA	++	++	++	++	?	++	NA	NA
Nakazono T et al.^15)^	Intra-rater	No	−	?	?	?	?	?	++	NA	NA
Nakazono T et al.^15)^	Inter-rater	No	−	?	?	++	++	?	++	NA	NA

Columns: (1) Were patients stable in the time between the repeated measurements on the construct to be measured? (2) Was the time interval between the repeated measurements appropriate? (3) Were the measurement conditions similar for the repeated measurements–except for the condition being evaluated as a source of variation? (4) Did the professional administer the measurement without knowledge of scores or values of other repeated measurement in the same patients? (5) Did the professional assign scores or determine values without knowledge of the scores or values of other repeated measurement in the same patients? (6) Were there any other important flaws in the design or statistical methods of the study? (7) For continuous scores, was an intraclass correlation coefficient (ICC) calculated? (8) For ordinal scores, was a (weighted) kappa calculated? (9) For dichotomous/nominal scores, was Kappa calculated for each category against the other categories combines? ++, very good; +, adequate; ?, doubtful; −, inadequate; COSMIN, COnsensus-based Standards for the selection of health Measurement INstruments; NA, not applicable.

**Table 3.** Results of bias risk assessment of studies on measurement error using COSMIN Risk of Bias

Study	Reliability type	Simultaneous	(1)	(2)	(3)	(4)	(5)	(6)	(7)	(8)
Beckerman et al.^30)^	Intra-rater	No	++	++	?	?	?	?	++	NA
Hiengkaew V et al.^29)^	Inter-rater	No	?	?	?	?	?	?	+	NA
Hsueh I et al.^28)^	Intra-rater	No	?	?	?	?	?	?	+	NA
Kim H et al.^27)^	Intra-rater	No	?	++	?	++	++	?	++	NA
Kim H et al.^27)^	Inter-rater	Yes	NA	++	++	++	++	?	++	NA
Nakazono T et al.^15)^	Intra-rater	No	−	?	?	?	?	?	?	NA
Nakazono T et al.^15)^	Inter-rater	No	−	?	?	++	++	?	++	NA

Columns: (1) Were patients stable in the time between the repeated measurements on the construct to be measured? (2) Was the time interval between the repeated measurements appropriate? (3) Were the measurement conditions similar for the repeated measurements – except for the condition being evaluated as a source of variation? (4) Did the professional administer the measurement without knowledge of scores or values of other repeated measurement in the same patients? (5) Did the professional assign scores or determine values without knowledge of the scores or values of other repeated measurement in the same patients? (6) Were there any other important flaws in the design or statistical methods of the study? (7) For continuous scores, was the standard error of measurement (SEM), smallest detectable change (SDC), limits of agreement (LOA) or coefficient of variation (CV) calculated? (8) For dichotomous/nominal/ordinal scores, was the percentage-specific (e.g. positive and negative) agreement calculated? ++, very good; +, adequate; ?, doubtful; −, inadequate; COSMIN, COnsensus-based Standards for the selection of health Measurement INstruments; NA, not applicable.

**Table 4.** Results of bias risk assessment of studies on responsiveness-criterion approach (i.e., comparison to a gold standard) using the COSMIN Risk of Bias checklist

Study	(1)	(2)	(3)
Pandian et al.^18)^	++	++	++

Columns: (1) For continuous scores, were correlations between changes in scores, or the area under the receiver operator characteristic (ROC) curve calculated? (2) For dichotomous scales, were sensitivity and specificity (changed vs unchanged) determined? (3) Were there any other important flaws in the design or statistical methods of the study? ++, very good; +, adequate; ?, doubtful; −, inadequate; COSMIN, COnsensus-based Standards for the selection of health Measurement INstruments.

## Discussion

This scoping review identified 5 MDC studies and one MIC study that estimated values for the FMA-LE motor scale. No late-subacute threshold was identified for the MDC and no acute or subacute threshold was identified for the MIC. Regarding the quality of the MDC studies, only one study received a “very good” or “adequate” rating on more than half of the checklist items, which indicated a high risk of bias in the study design. Regarding the quality of the MIC study, it received a “very good” rating on all checklist items, which indicated a low risk of bias.

### Difference of thresholds among disease phases regarding the MDC

The present study showed higher thresholds for the MDC in the chronic phase^27–30)^ than in the acute and early subacute phase^15)^. This result may be contrary to previous studies that investigated reliability or measurement error for motor-related function tools in stroke patients^52,53)^. Generally speaking, measurement errors can be considered to be greater in test-retest results during the acute phase, when the patient’s functional status is more likely to change. However, in reliability studies, the thresholds decrease when the interval between evaluations is shorter^13,54)^ or when the patients’ functional status is high^55)^.

In the articles included in this review, the acute and early subacute phase study^15)^ used a very short interval between evaluations (i.e., on the same day), whereas the chronic phase studies used longer intervals such as 1–3 weeks^27–30)^. In addition, the scores on the FMA-LE motor scale were higher in the acute and early subacute phase study than in the chronic phase studies. Based on these aspects, the thresholds in the chronic phase studies may be higher than that in the acute and early subacute phase study in this review. In reliability studies, researchers must assume that the patient remained unchanged in the interval between the two evaluations, so for the MDC, it might be necessary to pay more attention to the evaluation interval and the patient’s score than to the disease phase.

### Difference in thresholds between intra-rater and inter-rater reliabilities for the MDC

The intra-rater reliability thresholds in some studies^27,28,30)^ were higher than that of the inter-rater reliability in one study^29)^ in the chronic phase. Normally, the inter-rater reliability condition has more bias risk factors compared with the intra-rater reliability condition, because the different assessors rate patients independently. As a result, previous studies have reported that the thresholds of the inter-rater reliability in two studies were higher than that of the intra-rater reliability in other motor function assessments^40,56)^. In the articles included in this review, intra-rater reliability in some studies^27,28,30)^ used longer intervals between evaluations than inter-rater reliability in one study^29)^. This means that the bias from the duration of the evaluation interval may be larger than the bias from the difference between the evaluators.

### Difference in thresholds between the MIC and MDC

Disease phase, which can be used to compare both the estimation approach methods, was only included in chronic phase studies. Most of the results^28–30)^ were lower than the 6 points of the anchor-based approach, but 1 study was about 2 points higher than the anchor-based approach^27)^. The divergence of this result may be derived from the fact that the changes in scores between assessments can differ when receiving a different treatment intervention^57)^. Basically, a higher threshold value of the MIC than of the MDC is preferred^19,58)^. However, it is often difficult to make simple comparisons for the MIC because the research often uses different intervention content, intervention frequency, intervention amount, and evaluation interval.

### Assessment of the methodological quality of the MDC studies

Regarding the quality of the MDC, only one study^27)^ was rated as “very good” or “adequate” in more than half of the items on the reliability and measurement error checklists. In previous review studies of measurement properties of outcome tools, the overall quality can be taken as the lowest rating for each checklist^59,60)^. In the present study as well, it can be considered that there was a high risk of bias in the quality of the MDC. Specifically, the decline in ratings of the items “patients’ stability and time interval between the repeated measurements” and “similarity of measurement conditions between the repeated measurements” can be seen as common problems. Regarding the patients’ stability, sufficient time is needed to allow the patient to recover from fatigue experienced between repeated measurements and to allow the patient to return to the state before the first evaluation. Regarding the time interval in the acute or sub-acute phase, motor function can change in a few days. Therefore, researchers need to ensure that patients have not had any changes in the function to be measured with solid evidence or clear assumptions made by the medical professionals involved. Regarding the similarity of measurement conditions, researchers need to make sure that all equipment, preparatory actions, environmental conditions (e.g., a ward bed for rest or a training bed of relatively hard material), and processing protocols were the same in both measurements. These issues have been raised in other motor-related outcomes,^43,44)^ so researchers should be aware that these issues should be improved in future reliability investigation studies.

## Limitations

The main limitation of the present study is that there were differences in baseline FMA-LE scores due to the severity of motor hemiplegia, which affects MIC and MDC thresholds. The concepts of MIC and MDC were expected to differ according to the motor function level. Second, the influences of stroke type, age, sex, and onset severity were not evaluated. Third, only articles published in English were included, which may introduce publication bias in the scoping review. Given the burgeoning interest in this topic, more rigorous studies controlling for the effects of motor function severity and other confounding factors are warranted after more MIC and MDC studies have been published.

## Conclusions

This scoping review identified many gaps in the studies that estimated the MIC and MDC. No late subacute threshold was identified for the MDC and no acute or subacute threshold was identified for the MIC. The quality of the MIC studies was “adequate”. The quality of the MDC study has room for improvement in study design.

## Funding

Not applicable.

## Conflicts of Interest

The author declares no conflicts of interest.

## Supplementary Materials

Supplementary Material 1. Preferred Reporting Items for Systematic reviews and Meta-Analyses extension for Scoping Reviews (PRISMA-ScR) Checklist;

Supplementary Material 2. Search strategy;

Supplementary Material 3. Reliability, distribution, and anchor characteristics of FMA-LE of the included studies.
