# Supplementary material for: A Scoping Review of Minimal Important Change and Minimal Detectable Change of the Fugl-Meyer Assessment Lower Extremity Scale in Patients with Stroke
Source: Phys Ther Res. 2025 Jun 4;28(2):137–44. doi: 10.1298/ptr.E10324 (PMC12445363; doi:10.1298/ptr.E10324)
Supplement: Supplementary Material 2. — Search strategy; [file ptr-28-137-s02.pdf]

Supplementary material 2. Search strategy	
Datebases	Search strategy
PubMed	("Fugl-meyer"[Title/Abstract] OR "FMA"[Title/Abstract] OR "FMT"[Title/Abstract] OR "FM test"[Title/Abstract] OR "FM motor"[Title/Abstract]) AND (("MCID"[tiab] OR "minimal clinically important difference"[tiab] OR "SSD"[tiab] OR "subjectively significant difference"[tiab] OR "MID"[tiab] OR "minimal important difference"[tiab] OR "CS"[tiab] OR "clinical significance"[tiab] OR "CID"[tiab] OR "clinically important difference"[tiab] OR "CSC"[tiab] OR "clinically significant change"[tiab] OR "MIC"[tiab] OR "minimally important change"[tiab] OR "MDD"[tiab] OR "minimally detectable difference"[tiab] OR "SEM" OR "standard error of measurement"[tiab] OR "MDC" OR "minimum detectable change"[tiab] OR "SRD"[tiab] OR "smallest real difference"[tiab] OR "SDD"[tiab] OR "smallest statistically detectable difference" or "smallest detectable difference"[tiab] OR "LOA"[tiab] OR "limits of agreement"[tiab] OR "SDC"[tiab] OR "smallest detectable change"[tiab]) OR ((minimal[tiab] OR minimally[tiab] OR clinical[tiab] OR clinically[tiab]) AND (important[tiab] OR significant[tiab] OR detectable[tiab]) AND (change[tiab] OR difference[tiab])) OR (small*[tiab] AND (real[tiab] OR detectable[tiab]) AND (change[tiab] OR difference[tiab])) OR "meaningful change"[tiab] OR "psychometrics"[MeSH] OR psychometr*[tiab] OR clinimetr*[tw] OR clinometr*[tw] OR "outcome assessment (health care)"[MeSH]) AND hasabstract NOT (("addresses"[Publication Type] OR "biography"[Publication Type] OR "case reports"[Publication Type] OR "comment"[Publication Type] OR "directory"[Publication Type] OR "editorial"[Publication Type] OR "festschrift"[Publication Type] OR "interview"[Publication Type] OR "lectures"[Publication Type] OR "legal cases"[Publication Type] OR "legislation"[Publication Type] OR "letter"[Publication Type] OR "news"[Publication Type] OR "newspaper article"[Publication Type] OR "patient education handout"[Publication Type] OR "popular works"[Publication Type] OR "congresses"[Publication Type] OR "consensus development conference"[Publication Type] OR "consensus development conference, nih"[Publication Type] OR "practice guideline"[Publication Type]) OR ("animals"[MeSH Terms] NOT "humans"[MeSH Terms])Filters applied: Abstract, English, Humans

Web of Science	"Fugl-meyer" or "FMA" or "FMT" "FM test" or "FM motor" (topic) and "MCID" or "minimal clinically important difference" or "SSD" or "subjectively significant difference" or "MID" or "minimal important difference" or "CS" or "clinical significance" or "CID" or "clinically important difference" or "CSC" or "clinically significant change" or "MIC" or "minimally important change" or "MDD" or "minimally detectable difference" or "SEM" or "standard error of measurement" or "MDC" or "minimum detectable change" or "SRD" or "smallest real difference" or "SDD" or "smallest statistically detectable difference" or "smallest detectable difference" or "LOA" or "limits of agreement" or "SDC" or "smallest detectable change" or "meaningful change" or ((minimal OR minimally OR clinical OR clinically) AND (important OR significant OR detectable) AND (change OR difference)) OR (small* AND (real OR detectable) AND (change OR difference)) (topic) and English (language) and Articles（original article） and Engineering Biomedical or Sport Sciences or Dentistry Oral Surgery Medicine or Surgery or Radiology Nuclear Medicine Medical Imaging or Biotechnology Applied Microbiology or Engineering Electrical Electronic or Mathematical Computational Biology or Medical Informatics or Physics Applied or Polymer Science or Chemistry Physical or Engineering Multidisciplinary or Pharmacology Pharmacy or Physiology or Cell Biology or Chemistry Multidisciplinary or Computer Science Artificial Intelligence or Meteorology Atmospheric Sciences or Physics Condensed Matter or Psychiatry or Automation Control Systems or Biochemical Research Methods or Biophysics or Chemistry Analytical or Chemistry Applied or Computer Science Interdisciplinary Applications or Endocrinology Metabolism or Food Science Technology or Gastroenterology Hepatology or Gerontology or Toxicology or Tropical Medicine or Anatomy Morphology or Biochemistry Molecular Biology or Biology or Cell Tissue Engineering or Chemistry Medicinal or Chemistry Organic or Computer Science Cybernetics or Ecology or Energy Fuels or Engineering Chemical or Engineering Environmental or Engineering Manufacturing or Engineering Petroleum or Forestry or Marine Freshwater Biology or Materials Science Biomaterials or Materials Science Characterization Testing or Materials Science Composites or Mechanics or Nanoscience Nanotechnology or Nutrition Dietetics or Oceanography or Physics Atomic Molecular Chemical or Physics Mathematical or Plant Sciences or Telecommunications (excluded – Web of Science)

CINAHL	IN ("Fugl-Meyer Assessment" OR "Fugl-Meyer test" OR "Fugl Meyer scale" OR "FMA" OR "FM test" OR "FM motor" OR "FMT") AND MW ("physical sciences" OR "outcomes (health care)" OR "research measurement" OR "instrument by type" OR "functional assessment" OR "Psychomotor Performance" OR "Motor Skills Disorders" OR "Instrument Validation" OR "Reliability Evaluation" OR "instrument scaling" OR "instrument adaptation" OR "instrument construction")Filters applied: Abstract, English, Humans
