# Supplementary material for: A Scoping Review of Minimal Important Change and Minimal Detectable Change of the Fugl-Meyer Assessment Lower Extremity Scale in Patients with Stroke
Source: Phys Ther Res. 2025 Jun 4;28(2):137–44. doi: 10.1298/ptr.E10324 (PMC12445363; doi:10.1298/ptr.E10324)
Supplement: Supplementary Material 3. — Reliability, distribution, and anchor characteristics of FMA-LE of the included studies. [file ptr-28-137-s03.pdf]

Supplementary material 3. Reliability, distribution, and anchor characteristics of FMA-LE of the included studies

Author	Year	Country	Age (IQR)	Male (%)	Ischemic stroke (%)	Mean time after stroke onset
Beckerman, et al. [30)]	1996	Netherlands	58 (21–72)	33 (67)	36 (73)	37 (13–185) months
Hiengkaew V, et al. [29)]	2012	Thailand	63.5 ± 10.0	43 (70)	38 (62)	40.2 ± 34.3 months
Hsueh I, et al. [28)]	2008	Taiwan	57.9 ± 8.5	36 (60)	NA	NA
Kim H, et al. [27)]	2012	Korea	59.5 ± 12.4	26 (52)	21 (42)	2.4±1.8 years
Kim H, et al. [27)]	2012	Korea	59.5 ± 12.4	26 (52)	21 (42)	2.4 ± 1.8 years
Nakazono T, et al. [15)]	2022	Japan	74.4 ± 11.2	4 (40)	10 (100)	9.5 (7–10) days
Nakazono T, et al. [15)]	2022	Japan	69.0 ± 10.3	5 (50)	7 (70)	6.7 (5–7) days
Pandian, et al. [18)]	2016	India	44.2±12.8	42 (65)	31 (48)	16.4 ± 6.2 months

(Continued)

Author	Disease stage	Sample size	The FMA-LE total score	Reliability-tested		
				Simultaneous	Reliability type	Reliability score (95%CI)
Beckerman, et al. [30]	Chronic	49	17.7±4.5	No	Intra-rater	ICC=0.86 (NA)
Hiengkaew V, et al. [29]	Chronic	61	NA	No	Inter-rater	ICC=0.94 (0.89–0.98)
Hsueh I, et al. [28]	Chronic	60	22.1±5.4	No	Intra-rater	ICC=0.95 (0.91–0.97)
Kim H, et al. [27]	Chronic	50	22.2±7.9	No	Intra-rater	ICC=0.93 (0.89–0.96)
Kim H, et al. [27]	Chronic	50	21.2±8.1	Yes	Inter-rater	ICC=0.86 (0.76–0.93)
Nakazono T, et al. [15]	Early subacute	10	25.5±7.7	No	Intra-rater	ICC=0.98 (0.94–0.99)
Nakazono T, et al. [15]	Acute	10	26.4±7.9	No	Inter-rater	ICC=0.98 (0.92–0.99)
Pandian, et al. [18]	Chronic	65	20.2±4.3	NA	NA	NA

(Continued)

Author	Distribution-based analysis		Anchor-based analysis	
	SEM	MDC	Patient-driven outcome	Other-driven outcome
Beckerman, et al. [30)]	1.76	4.87	NA	NA
Hiengkaew V, et al. [29)]	1.29	3.57	NA	NA
Hsueh I, et al. [28)]	1.38	3.80	NA	NA
Kim H, et al. [27)]	2.88	7.98	NA	NA
Kim H, et al. [27)]	2.15	5.96	NA	NA
Nakazono T, et al. [15)]	0.45	1.24	NA	NA
Nakazono T, et al. [15)]	1.17	3.23	NA	NA
Pandian, et al. [18)]	NA	NA	6.00	6.00

Abbreviations: FMA-LE, Fugl-Meyer Assessment Lower Extremities; IQR, interquartile range; ICC, intraclass correlation coefficient; 95%CI, 95%Confidence interval; NA, Not Applicable; SEM, standard error of measurement; MDC, minimal detectable change.

Note: (A), intra-rater reliability study; (B), inter-rater reliability study; acute, ≤ 2 weeks from stroke onset; acute, ≤ 7 days from stroke onset; early subacute, ≤ 7days-3 months from stroke onset; late subacute, ≤ 3-6 months from stroke onset; chronic, >6 months from stroke onset; MDC=1.96*SEM*√2; Other-driven outcome, Functional Ambulation Classification
